# A Security Information Transmission Method Based on DHR for Seafloor Observation Network

**DOI:** 10.3390/s24041147

**Published:** 2024-02-09

**Authors:** Fei Ying, Shengjie Zhao, Jia Wang

**Affiliations:** 1 College of Electronic and Information Engineering, Tongji University, Shanghai 201804, China; 23yingfei@tongji.edu.cn; 2 Key Laboratory of Embedded System and Service Computing, Ministry of Education, Shanghai 201804, China; 3 School of Software Engineering, Tongji University, Shanghai 201804, China; 4 Engineering Research Center of Key Software Technologies for Smart City Perception and Planning, Ministry of Education, Shanghai 200003, China; 5 School of Advanced Technology, Xi’an Jiaotong-Liverpool University, Suzhou 215123, China

**Keywords:** DHR architecture, seafloor observation network, privacy security

## Abstract

A seafloor observation network (SON) consists of a large number of heterogeneous devices that monitor the deep sea and communicate with onshore data centers. Due to the long-distance information transmission and the risk of malicious attacks, ensuring the integrity of data in transit is essential. A cryptographically secure frame check sequence (FCS) has shown great advantages in protecting data integrity. However, the commonly used FCS has a collision possibility, which poses a security risk; furthermore, reducing the encryption calculation cost is a challenge. In this paper, we propose a secure, lightweight encryption scheme for transmitted data inspired by mimic defense from dynamic heterogeneous redundancy theory. Specifically, we use dynamic keys to encrypt a data block and generate multiple encrypted heterogeneous blocks for transmission. These continuously changing encrypted data blocks increase the confusion regarding the original encoded data, making it challenging for attackers to interpret and modify the data blocks. Additionally, the redundant information from the multiple blocks can identify and recover tampered data. Our proposed scheme is suitable for resource-constrained environments where lightweight encryption is crucial. Through experimental demonstrations and analysis methods, we determine the effectiveness of our encryption scheme in reducing computational costs and improving security performance to protect data integrity.

## 1. Introduction

**Figure 1:**
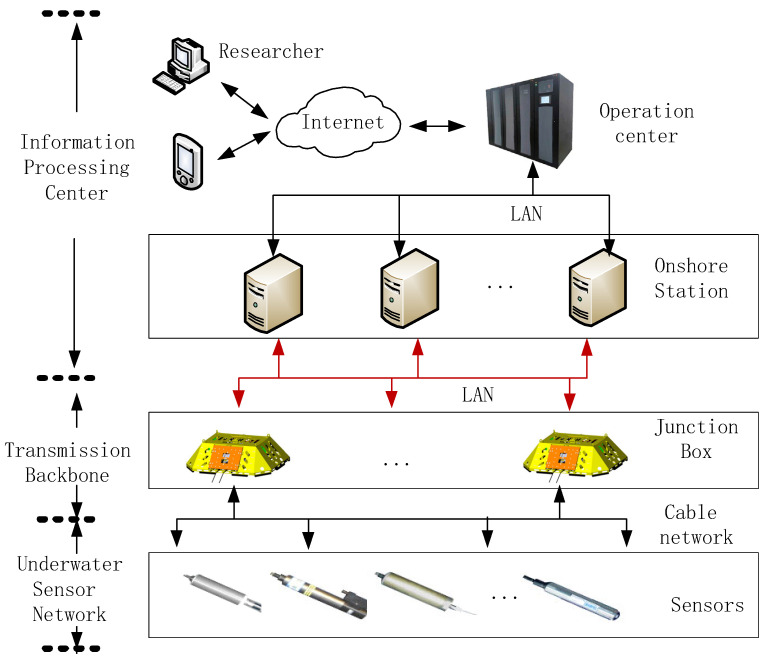
Information monitoring system in seafloor observatory network.

A seafloor observation network (SON) is an emerging platform for human observation of the ocean. A SON consists of various wire-connected seafloor sensors working collaboratively to monitor vast deep-sea environments (Figure 1). As a permanent infrastructure, cabled SON can provide abundant power and broad bandwidth communication [1]. It enables all-weather, in situ, continuous, real-time, and high-precision observation of the ocean from the sea floor to the sea surface, which is crucial to the development of marine science [2].

A SON requires the collection, storage, transmission, and processing of massive amounts of marine data from sensors to operation centers. To ensure efficient transmission, junction boxes serve as relay nodes, processing fragmented data from cable-connected sensors into structured data blocks and transmitting them to onshore stations. However, long-distance data transmission makes unintentional (e.g., packet loss) and intentional (e.g., tampering attacks) errors or changes to the data more likely, which can be difficult to detect [3]. Consequently, mechanisms are needed to ensure secure information transmission from the seafloor to the shore. Notably, onshore stations are designed with sufficient buffer and computing resources to store received data and handle altered or missing data. Therefore, the security of information transmission in SONs can be considered within the integrity of each individual data block inside a packet over long distances.

**Figure 2:**
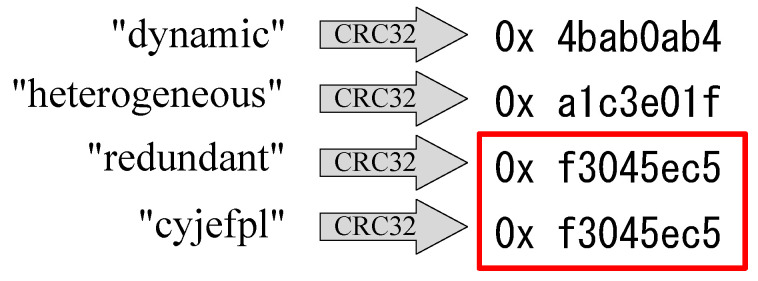
Collision example under CRC check (The CRC checksum values of ‘redundant’ and ‘cyjefpl’ are identical).

A frame check sequence (FCS), which adds redundancy or additional information to data blocks, is a common method for checking data integrity and detecting errors or changes in received data blocks. The three most frequently used techniques for generating FCS values are watermarking schemes, cyclic redundancy check (CRC), and cryptography algorithms [4]. Watermarking schemes offer lightweight data integrity schemes by inserting a secret piece of information, called a watermark, to detect changes in the original data stream [5]. These methods require redundant bits in the data to embed the watermark, which could be a weakness if the underwater sensors do not support modification of sent data blocks. Message authentication codes (MACs) [6] require the sender and receiver to share a secret key to verify the message’s integrity. However, these mechanisms work well only within medium-scale networks [7], as SONs consist of numerous sensors and relay nodes, and the vast data transfer might render the key unavailable. Cryptographic CRC checksums are another common way to secure data integrity with minimal extra resources [8]. As shown in Figure 2, CRC is susceptible to collisions. Considering the large-scale data transmission required by SONs, it is impossible to avoid CRC collisions, leading to security risks due to data integrity issues. In light of the aforementioned challenges and considerations, the core issue we aim to address in this paper pertains to ensuring the integrity of transmitted data. Specifically, we focus on managing the challenges posed by large volumes and the need for timely handling of delay-sensitive data. Our objective is to safeguard the data transmitted between seafloor sensors and onshore operation centers within SONs, thereby preventing potential threats such as tampering and unauthorized data access.

In this paper, we present an efficient and secure method for information transmission within a SON. Our approach concentrates on ensuring the secure transmission of data between junction boxes and onshore stations. The junction box compiles data from underwater sensors and constructs a secure data block for transmission to the onshore station. We employ a dynamic heterogeneous redundant (DHR) framework as a security measure, utilizing heterogeneity and redundancy to defend against various attack types. This framework is applicable to numerous applications, such as computer networks, distributed systems, and cybersecurity defenses [9]. Inspired by the DHR-based active defense framework, we encrypt data blocks with dynamic keys and generate multiple encrypted heterogeneous data blocks for transmission. These encrypted data blocks, as variants of the original data block, are expected to be decoded at the receiver with the same content. Inconsistency in the decoded content implies that the received data block has been altered. The redundant encrypted multi-blocks enhance security by increasing the attacker’s complexity since they cannot interpret the data using a single encrypted block. The main contribution of the paper can be summarized as follows:

1. We present a novel approach to safeguarding data transmission within a SON by utilizing a DHR framework. Our method’s simplicity and low computational complexity make it well-suited for deployment in SON devices with limited computational capabilities. To the best of our knowledge, this is the first instance of employing a DHR framework for this purpose;
2. We introduce an active defense framework that uses dynamic key encryption to encrypt data blocks and generates heterogeneous data blocks during transmission. This method significantly increases the difficulty for attackers trying to decipher the information, as a single encrypted block is insufficient for interpretation, thus enhancing the overall security of data integrity;
3. Experimental results provide evidence that the proposed framework effectively defends against data tampering and data-stealing attacks within a SON environment.

The paper is structured as follows: In Section 2, we introduce the structure of a SON and discuss the associated security risks. Section 3 provides a comprehensive review of the related works that serve as the foundation for our proposed method. In Section 4, we delve into the problem formulation and provide insights into the motivations behind our research. Section 5 is dedicated to the detailed explanation of our proposed method and includes an in-depth analysis of its performance. To validate the effectiveness of our approach in enhancing security defense, we present experimental results in Section 6. In Section 7, we summarize our research contributions and conclude the paper. Furthermore, we explore potential directions for future research in this field.

## 2. Background

### 2.1. Seafloor Observation Network

**Figure 3:**
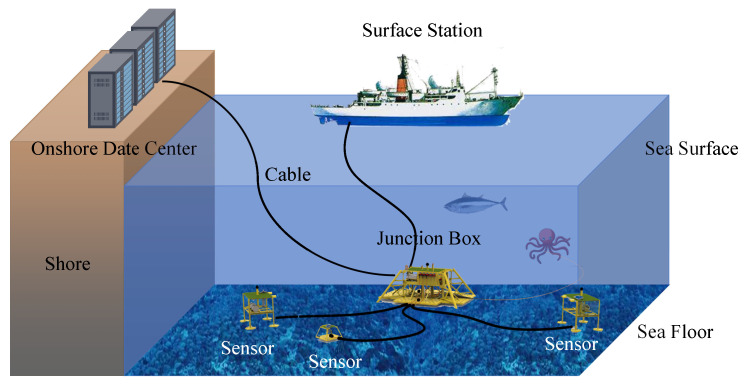
The illustration of a seafloor observation network.

As shown in Figure 3, a SON consists of both surface components (i.e., onshore data centers, surface stations) and underwater components (i.e., junction boxes and sensors). It enables long-term, large-scale monitoring of deep-sea regions. Specifically, the surface components supply power to the underwater components via optical cables and analyze the collected data. For safety purposes, the control unit at an onshore data center processes the data and automatically cuts off power when the warning system detects abnormal values. At the same time, the junction box in the underwater components converts high voltage to medium voltage, providing power to the underwater sensors and transmitting the collected information to the land station [10]. Common underwater sensors include acoustic Doppler current profilers (ADCP), hydrophones, conductivity–temperature–depth (CTD) sensors, and ocean-bottom seismographs (OBS). Numerous underwater sensors connect to a junction box in linear, tree-like, or ring-like configurations, forming an underwater sensor network.

### 2.2. Security Risks in SONs

It is important to note that SONs face several security risks. Cable providers may introduce backdoors or embed monitoring equipment and triggers in cable components before deployment [11]. Unauthorized or malicious use of these interfaces can lead to data leakage during transmission. Additionally, network management systems typically depend on HTTP or TCP/IP protocols for connections, which makes it easy for attackers to intercept protocol packets and analyze or obtain data information [12]. Attackers may also gain control over sensor nodes to steal or tamper with sensitive data [13]. Altered data could cause system failures, resulting in power cutoffs or fault isolation at onshore data centers, thereby disrupting continuous underwater environmental observations. Moreover, data leaks or tampering could pose serious threats to ocean observations and lead to critical decision-making errors. These significant risk concerns in SONs have hindered the advancement of seabed scientific researches.

## 3. Related Works

In this section, we review the methods to ensure data integrity and DHR applications that are closely related to our work.

### 3.1. Methods for Ensuring Data Integrity

Data integrity refers to the accuracy, validity, and consistency of information within a system. When transmitting data, especially over an unstable media (e.g., deep sea environment), several potential security issues arise, such as physical failure and malicious tampering. Ensuring data integrity is crucial to prevent data contamination, fraudulent data injection, and data manipulation [14]. Several technologies, including error-detecting codes, cryptography algorithms, arbitration schemes, and watermarking schemes, are frequently employed to address data integrity issues [15].

Error-detecting codes are widely used techniques in both wired and wireless networks, ensuring that only correctly marked frames are forwarded to higher-level communication protocols, while frames with errors are discarded. CRC, checksums, and MAC are a few examples of error-detecting codes. Among these, CRC stands out as particularly effective, employing binary division instead of addition. Standardized polynomials, such as CRC-16 and CRC-32, are common variants of CRC; however, when selecting a specific CRC polynomial, it is crucial to consider the trade-off between security and computational cost [8]. In the field of data integrity, several methods use CRC to ensure the reliability and accuracy of data. For instance, Chen et al. [16] reduced the overall cost of the prevention and repair stage in distributed systems by implementing redundant error correction codes and network coding. Yu et al. [17] ensured data integrity by using identity CRC, providing an effective way to protect privacy data. Similarly, Ateniese et al. [18] employed a forward error-correcting code to enhance the performance of data processing frameworks. Despite their advantages, encoding-based methods can lead to increased computational costs and reduced running efficiency when using large security keys and blocks.

Encrypting data during transmission can protect its integrity. Various encryption methods exist, including symmetric encryption, asymmetric encryption, and hash mapping [19]. Symmetric encryption employs a single key for both encryption and decryption, while asymmetric encryption uses a pair of public and private keys for enhanced security. Hash mapping transforms data into a fixed-sized hash through mathematical methods. Common encryption methods include the Caesar cipher [20], Data Encryption Standard (DES) [21], Triple Data Encryption Standard (3DES) [22], Advanced Encryption Standard (AES) [23], and BlowFish [24]. However, when data is transmitted through encryption, the security of the encrypted data becomes vulnerable if the key is lost or stolen.

Arbitration is another method for protecting the accuracy and completeness of data through third-party verification. Data arbitration can be categorized into the following two main security models: provable data possession (PDP) and proof of retrievability (PoR). PDP includes static and dynamic schemes. The static PDP scheme focuses on protecting the security of confidential data, but it lacks the capability to restore lost data [25]. Meanwhile, the dynamic PDP scheme focuses on dynamic data updates, enabling recovery of some lost data by incorporating error-correcting codes [26]. However, the arbitration process faces challenges concerning privacy data breaches and authentication of third-party identities.

Watermarking-based techniques aim to provide lightweight solutions for data integrity and authentication, which embeds a secret piece of information, known as a watermark, into the original data streams to detect any alterations. In recent years, they have been widely used in data transmission to prevent private information from being illegally obtained [27]. Al-Shayea et al. [28] proposed a new watermarking method based on the use of orthogonal families to withstand various types of attacks. Ferdowsi et al. [29] applied deep learning technology to dynamic watermarking to identify attack threats in the Internet of Things. However, attackers can easily decipher watermarking methods, and the cost of computation remains high. Furthermore, several watermarking techniques require the addition of extra bits in the data stream to embed the watermark, posing a vulnerability if the transmission does not support the data distortions.

### 3.2. DHR Architecture and Applications

**Figure 4:**
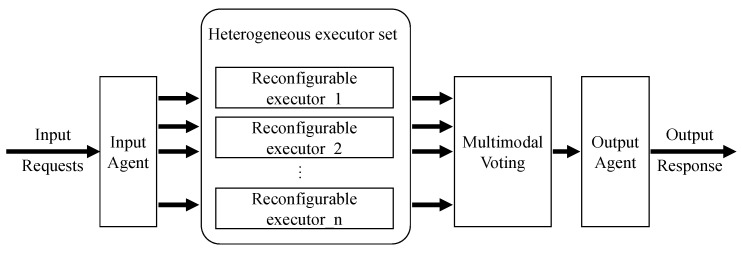
The overview of DHR architecture.

DHR architecture is an endogenous security technique, as depicted in Figure 4. Within this framework, the input agent plays a crucial role in distributing input requests to a diverse set of heterogeneous redundant executors, each responsible for independent processing. Subsequently, the processing results undergo a multimodal voting process, and only the consistently matching results are chosen as the final output. This approach significantly reduces the risk of security weaknesses and vulnerabilities being exploited, thereby ensuring the trustworthiness of the system results. This architecture is widely adopted in the domain of endogenous security. Wei et al. [30] proposed a mimic web application security technology based on the DHR architecture, which makes it difficult for attackers to maintain continuous control and access after a successful attack. Yu et al. [31] successfully applied the DHR architecture to industrial network security, effectively increasing the difficulty of exploiting backdoors, such as paralysis, rule tampering, and information theft. Furthermore, DHR architecture’s adaptability is evident from its successful implementation in various domains, including the Internet of Vehicles [32] and edge networks [33]. These real-world deployments have demonstrated the versatility and effectiveness of DHR architecture in guarding against potential security threats.

## 4. Preliminaries

### 4.1. Notations and Problem Formulation

Let $E=\{E_{1}\left(\right),E_{2}\left(\right),\cdots ,E_{n}\left(\right)\}$ be a set of *n* mapping functions. The *i*-th sender encrypts a message *I* using a mapping function $E_{i}\left(\right)\in E$, resulting in ciphertext $E_{i}\left(I\right)$ transmitted to the receiver. We use $A_{send}$ to denote the information space and $A_{recv}^{E_{i}}$ is the encrypted space based on $E_{i}\left(\right)$. The receiver decrypts the ciphertext using the inverse function $E_{i}^{-1}\left(\right)$. The above process satisfies the following properties:

- Invertibility: For any $I\in A_{send}$, there exists a unique message $I^{\prime }\in A_{recv}$ such that $E_{i}\left(I\right)=I^{\prime }$ and $E_{i}^{-1}\left(I^{\prime }\right)=I$;
- Redundancy: For any $I\in A_{send}$, where $E_{i}\left(\right)\neq E_{j}\left(\right)$, there exists the encrypted information $E_{i}\left(I\right)$ = $E_{j}\left(I\right)$;
- Uniqueness: For any $I^{\prime }\in A_{recv}$ such that $E_{i}^{-1}\left(\right)\neq E_{j}^{-1}\left(\right)$, then decoded information $E_{i}^{-1}\left(I^{\prime }\right)\neq E_{j}^{-1}\left(I^{\prime }\right)$.

Our objective is to protect data integration by encrypting information using the above mapping functions. For clarity, we summarize the frequently used notations in Table 1:

### 4.2. Security Assumptions

The security of the proposed scheme is based on the following two attack problems:

- Data tampering attack: Refers to unauthorized changes made to data blocks while they are being transmitted. This attack is considered successful if the attacker is able to modify the data without detection by the system;
- Data stealing attack: Occurs when attackers gain access to a network and steal sensitive data while it is in transit.

### 4.3. Motivation

The limited number of mapping functions in E poses a security risk for SONs as they are rarely updated in reality. Attackers can exploit unknown vulnerabilities to launch brute-force attacks and guess the mapping function used for data transmission. To prevent such attacks, we propose equipping random perturbation parameters λ to the encryption result of mapping functions, denoted as $E_{i}\left(\cdot ;\lambda \right)$, where λ is the key randomly selected from a pool Λ.

## 5. Methodology

In this section, we introduce the system model and its application in SONs for ensuring information transmission integrity.

### 5.1. System Model

**Figure 5:**
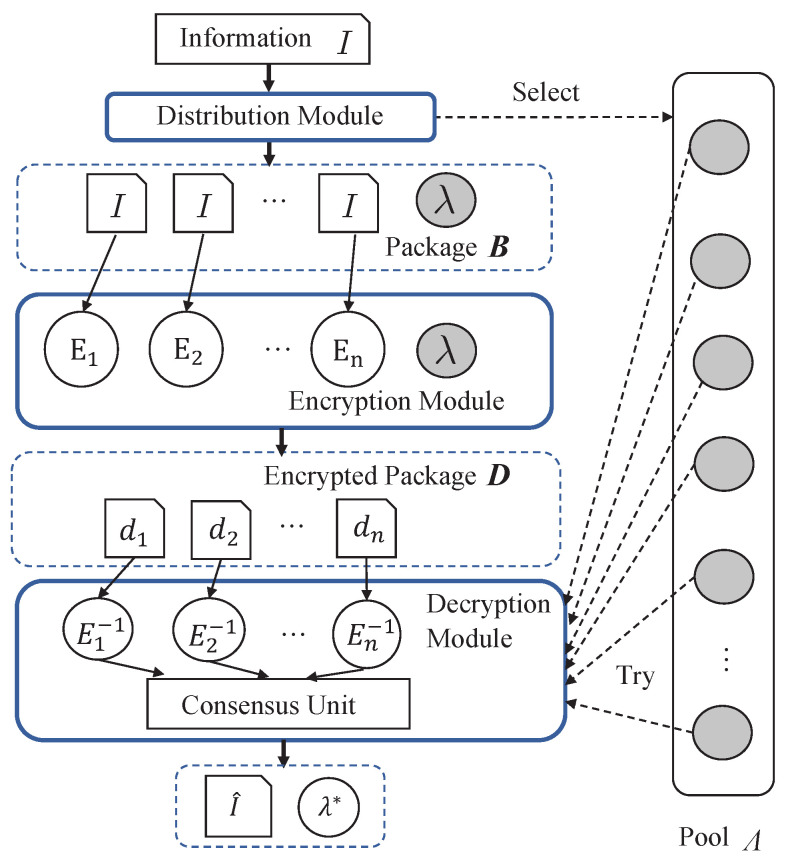
The illustration of system model.

The DHR architecture is a security approach that leverages heterogeneity and redundancy to protect systems against various types of attacks. As shown in Figure 5, the system model of a DHR-based security framework involves the following three main entities: distribute module, heterogeneous encryption module, and decryption module.

Distribution module takes an input *I* and generates a package B=[I,λ] by duplicating *I* into a set $I=\{I_{1},I_{2},\ldots ,I_{n}\}$ of *n* identical copies, and selecting a random key λ from a pool Λ. The resulting package B is then forwarded to the encryption unit for further processing.

Encryption module comprises *n* encryption units, each utilizing a unique mapping function $E_{i}\left(\right)\in E$ and the received key λ to encrypt the *i*-th element of I. The resulting encrypted ciphertext package is denoted as D, where $d_{i}\in D$ represents the encryption of the *i*-th element of I using the *i*-th encryption unit and the received key, i.e., $d_{i}=E_{i}\left(I_{i},\lambda \right)$.

Decryption module consists of *n* decryption units and a consensus unit. Each decryption unit attempts to decrypt the ciphertext package D using the corresponding decryption mapping function by trying each key in a pool Λ. Specifically, for a given key λ, the output of the *i*-th decryption unit is denoted as ${\hat{I}}_{i}=E_{i}^{-1}\left(d_{i},\lambda \right)$, where $E_{i}^{-1}\left(\right)$ is the inverse function of the encryption function used to encrypt the data $d_{i}$. The consensus unit then compares the outputs from all decryption units and selects the key $\lambda^{*}$ that yields consistent outputs across all units. This is performed by maximizing the consensus function Γ over all keys in the pool: $\lambda^{*}={\mathrm{argmax}}_{\lambda \in \Lambda }\Gamma \left(E_{1}^{-1}\left(d_{1},\lambda \right),E_{2}^{-1}\left(d_{2},\lambda \right),\cdots ,E_{n}^{-1}\left(d_{n},\lambda \right)\right)$, where Γ(·) is a consensus function that evaluates the congruence of outputs generated by each decryption unit, given a specific key λ. The outcome of Γ(·) is a quantified score that reflects the degree of consensus among the decryption outputs. Consequently, the selected key $\lambda^{*}$ is responsible for maximizing this consensus score. The final decrypted output is attained by applying the identified optimal key $\lambda^{*}$ to one of the decryption units that produces the consensual output.

### 5.2. Application in SONs

**Figure 6:**
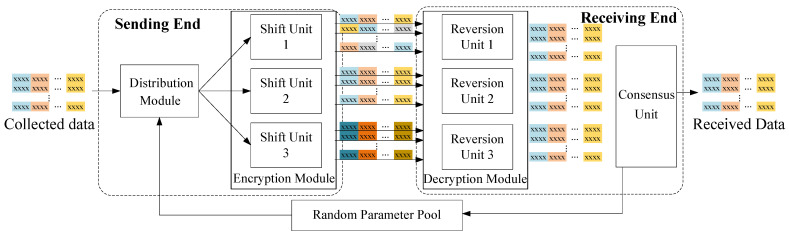
The systematic implementation of a seafloor observation network. (The data from the same sensor are shown in the same color).

In this section, we detail the implementation of our proposed security method for transmitting information in SONs. As illustrated in Figure 6, our system architecture is composed of the sending end, located on the junction boxes, and the receiving end, positioned at the onshore station. The sending end comprises a distribution module (DiM) and an encryption module (EnM) composed of three heterogeneous shift units. These components play a crucial role in ensuring the security of the transmitted information. On the other hand, the receiving end consists of three reversion units functioning as the decryption module (DeM) and a consensus unit. Their primary task is to recover the transmitted information by decrypting the received data. To provide a deeper understanding of our system architecture and its components, we present the details of each module in the subsequent subsections.

#### 5.2.1. Distribution Module

**Figure 7:**
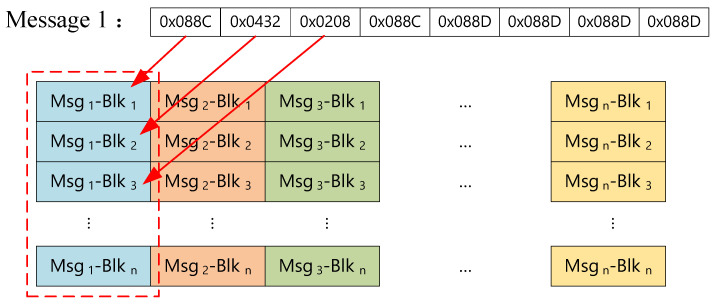
Message structure and stacked packets. (The data from the same sensor are shown in the same color).

SONs organize sensor data into messages consisting of *n* blocks of 4-bit hexadecimal numbers. In our framework, these messages are initially transmitted to a DiM, which receives *n* blocks at a time. The DiM then applies the stacking blocks method [34] to combine messages from *m* sensors and reorganize them into the stacked packets denoted by D. As illustrated in Figure 7, data blocks from the same sensor are listed in the same column. To enhance security, the stacked packets are concurrently dispatched to multiple shift units within EnM, along with the temporary dynamic key λ selected from a key pool Λ.

#### 5.2.2. Encryption Module

**Figure 8:**
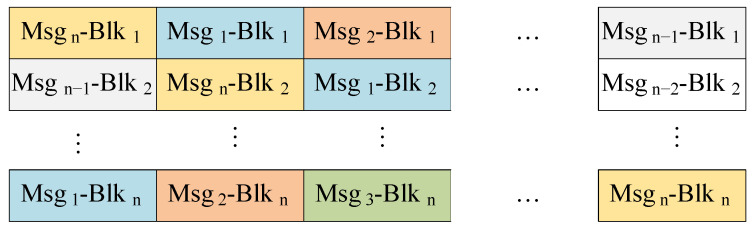
The data package encryption with the horizontal strategy (λ=a).
- Vertically translation strategy. The second shift strategy involves vertically translating the data blocks, where the columns of each block are preserved while the rows are shuffled using encryption parameters λ to produce the encrypted data packet $D^{\prime }$. For a given data block $d_{ij}$ in the stacked data packet *D*, it will be shifted to position $d_{i^{\prime },j^{\prime }}$, where $i^{\prime }$ and $j^{\prime }$ are determined based on following equation:
  (2) $$\left\{\begin{array}{cc} i^{\prime } & =(i+j+\lambda -a)\;\mathrm{mod}\;n\\ j^{\prime } & =j, \end{array}\right.$$
  where *i* and *j* represent the row and column of a data block in the original data packet, while $i^{\prime }$ and $j^{\prime }$ correspond to the row and column of the data block in the translated data packet. When λ equals *a*, the resulting vertically translated data packet is illustrated in Figure 9;

To minimize energy consumption during package encryption in the junction box and ensure cost-effectiveness for practical DHR-based applications [35], we employ a three-degree redundancy approach. Our encryption module comprises three heterogeneous shift units that use distinct shift strategies to transform data packet D into corresponding $D_{1}$, $D_{2}$, and $D_{3}$. Specifically, the three shift strategies are as follows:

- Horizontal strategy. For the data packet D, we shuffle the columns of the data blocks using encryption parameters λ while keeping the rows of each data block. This forms an encrypted data packet $D_{1}^{\prime }$, where data block $d_{ij}\in D$ will be translated to the position $d_{i^{\prime },j^{\prime }}$, where $i^{\prime }$ and $j^{\prime }$ satisfy the following conditions:
  (1) $$\left\{\begin{array}{cc} i^{\prime } & =i\\ j^{\prime } & =(i+j+\lambda -a)\;\mathrm{mod}\;n. \end{array}\right.$$
  where *a* a is a natural number constant. When λ sets *a*, the data packet after horizontal translation is shown in Figure 8;

**Figure 9:**
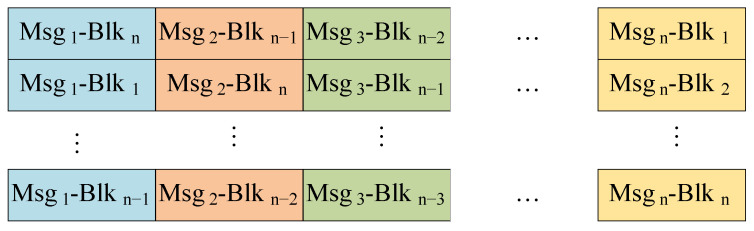
The data package encryption with the vertically strategy (λ=a).
- Numerical strategy. This strategy involves using a parameter λ to add a translation offset to the binary representation of the numerical value of each data block in data packet D. Since the information collected by the seabed observation sensor is comprised of 4-bit hexadecimal numbers, the data block $d_{ij}$ in D is transformed into $d_{i^{\prime },j^{\prime }}$ using the following formula:
  (3) $$d^{\prime }ij={\left(\left({\left(dij\right)}_{10}+i+j+\lambda -a\right)\;\mathrm{mod}\;16^{4}\right)}_{16},$$
  where ${\left(\cdot \right)}_{10}$ denotes decimal conversion, and ${\left(\cdot \right)}_{16}$ denotes hexadecimal conversion. The encrypted packet employing the numerical strategy is illustrated in Figure 10.

**Figure 10:**
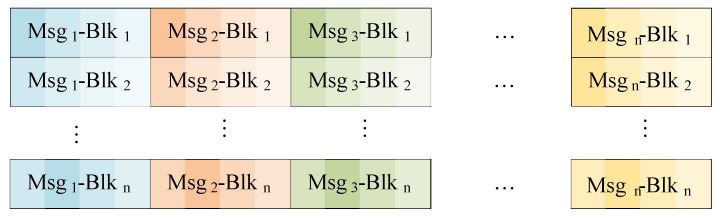
The data package encryption with the numerical strategy.

After undergoing processing by the heterogeneous encryption unit, the original data packet D is partitioned into the following three distinct packets: $D_{1}$, $D_{2}$, and $D_{3}$, which are then transmitted through the cable to the onshore data center or surface station. The integration of redundant shift operations effectively hinders attackers from deducing the original data values even if they intercept the transmission and possess prior knowledge of the collection process. This significantly raises the difficulty level for attackers attempting to steal authentic sensing data.

#### 5.2.3. Decryption Module

The DeM is located at the onshore station and is responsible for decrypting the received encrypted package. It tries different shifting parameters $\lambda^{\prime }$ from the pool Λ and uses the corresponding reverse rules to shift each data block in the package. By comparing the consistency of the three restored data blocks, the parameter $\lambda^{*}$ used at the encryption module can be determined, and the transmitted packet can be decrypted. The decryption process is shown in Algorithm 1.
**Algorithm 1** The decryption process.1:**function** Decryption($D_{1}$,$D_{2}$,$D_{3}$, Pool Λ)2:     **for** λ∈Λ **do**3:           $D_{1}^{\prime }$← horizonBack( $D_{1},\lambda$); /∗Reversed horizontal translation strategy;/∗4:           $D_{2}^{\prime }$← verticalBack( $D_{2},\lambda$); /∗Reversed vertical translation strategy;/∗5:           $D_{3}^{\prime }$← numBack( $D_{3},\lambda$); /∗Reversed numerical translation strategy;/∗6:           c ← consistency($D_{1}^{\prime },D_{2}^{\prime },D_{3}^{\prime }$); /∗Score the level of agreement;/∗7:           **if** $\max_{c}<c$ **then**8:                $\max_{c}\leftarrow c$, $\lambda^{*}\leftarrow \lambda$ /∗Get $\lambda^{*}$ that maximizes *c*;∗/9:      D← horizonBack( $D_{1},\lambda^{*}$) or D← verticalBack( $D_{2},\lambda^{*}$) or D← numBack( $D_{3},\lambda^{*}$)10:    **return** D

If a single sensor data is transmitted with an error or under a tampering attack, the encryption feedback controller is triggered, prompting the *distribution module* to select a new random parameter $\lambda^{\prime }$ from the pool for *encryption module* to shift the data packet for the upon the arrival data package, causing the translation rules of each data packet to change. This renders the previously observed data pattern unusable for the attacker, preventing them from continuing the attack experience. In general, the randomness of the new key $\lambda^{\prime }$ selected from the parameter pool Λ avoids an attacker launching a tampering attack. Thus, we use Equation (4) to quantify the randomness of the parameter key selection.

(4) $$H\left(\Lambda \right)=-\sum_{\lambda_{i}\in \Lambda }p\left(\lambda_{i}\right)log\left(p\left(\lambda_{i}\right)\right),$$

where $p(\lambda_{i})$ to represent the probability of obtaining the key $\lambda_{i}$. When each key in the emulation parameter pool has the same probability of selection, H(Λ) reaches its maximum value, indicating that the randomness of the emulation parameter selection is highest and the defense effect of the emulation-based data security system is best. The feedback controller can also defend against replay attacks.

### 5.3. Security Analysis

This subsection analyzes the behavior of our framework in the presence of an attack. Figure 6 illustrates a sequence of encrypted information packages transmitted through SONs. Throughout this subsection, we use the following notation: $D_{1},D_{2},D_{3}$ represents the encrypted stacked packets through *Shift Units* through horizontal, vertical, and numerical strategies, respectively. $d_{i,j}^{c}$ refers to the *i*-th data block belonging to the *j*-th sensor in the *c*-th stacked packet, where c∈{1,2,3}.

To decrypt encrypted packets, an attacker needs to know the random parameters used to encrypt data packets at the current moment. In other words, an attacker can decrypt encrypted packets at any time by choosing one parameter. Its success probability is constant, and it does not rise as the attacker’s data collection increases. In contrast, when using techniques like hash functions, the probability of success for the attackers rises as they gather more data.

#### 5.3.1. Tampering Packet

If the $d_{i,j}^{1}$ block is tampered with, the modifications affect some bytes in $d_{i,j}^{1}$. In this case, an integrity error is detected through the consensus unit at the receiving end. This error is detected because the output of the three reversion unit at $d_{i,j}$ is not the same. Therefore, this data block can be recovered if the output from the two remaining reversion unit outputs are identical at this position. Otherwise, retransmissions are requested for resending the $d_{i,j}^{1}$ values where there are consensus errors. If the maximum number of attempts is reached, the $d_{i,j}^{1}$ block is discarded.

#### 5.3.2. Data Theft

In this scenario, attackers listen to the data information transmitted in the LAN and analyze the collected information to obtain real data. As the values at the data block $d_{i,j}$ are changed over time, for example, at time $t_{a}$, the value at the position of the *i*-th data block of the *j*-th sensor is $d_{i,j}^{1}$, and at time $t_{b}$, the value at that position is $d_{i+x,j+y}^{1}$. This prevents attackers from obtaining the genuine sensor data by relying solely on the absolute packet positions. On the other hand, at different times, the distance between the real data reflected by $d_{i,j}^{1}$ and $d_{i+1,j}^{1}$ is different, which hinders attackers from extracting sensitive information using relative packet positions.

## 6. Implementation and Evaluation

This section outlines the hardware and software utilized in the implementation of the proposed schemes. Then, the experimental findings are discussed.

### 6.1. Environmental Setup

**Figure 11:**
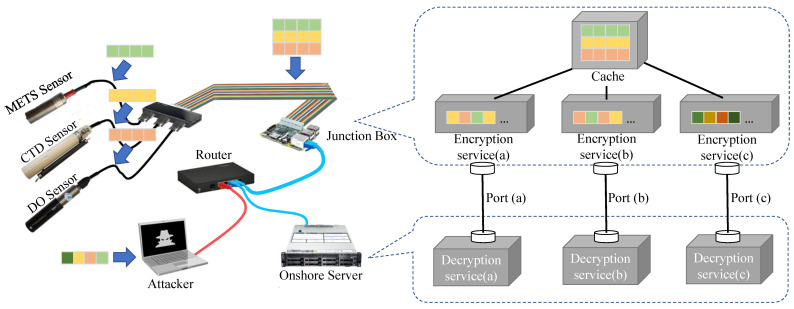
The experimental environment.

We conduct our experiments in the environment as shown in Figure 11. The hardware test-bed includes a METS sensor, a CTD sensor, and a DO sensor connected to a Raspberry Pi (i.e., junction box) that encrypts the sensing data and transmits it via cable to a ThinkSystem (i.e., onshore sever). The information is subsequently decoded by the onshore server. To attack the system, the attacker uses the router to obtain access to the network. Table 2 provides the details of the environment and configuration used in the experiment. The environment sensors are connected to the Raspberry Pi (i.e., junction box) through the RS422 serial port. The Raspberry Pi (i.e., junction box) is linked with the ThinkSystem (i.e., onshore sever) through the RJ-45 interface to form an Ethernet LAN. Additionally, we simulate an attacker who can access the network through a router and carry out attacks. Typically, the data-stealing attack is capable of capturing data packets transmitted in the LAN, while the data tampering attack could modify data packets transmitted in the LAN to deceive the receiver.

### 6.2. Simulated Man-in-the-Middle

**Figure 12:**
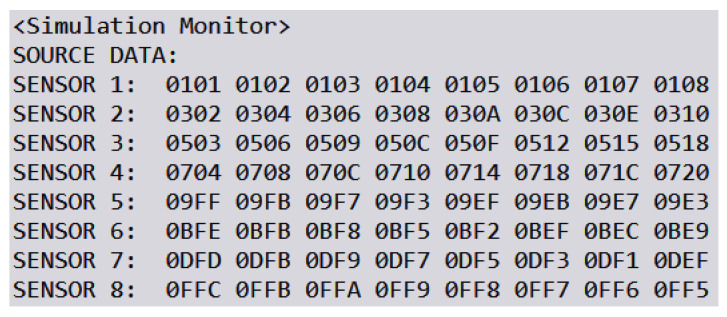
Data collected by simulated sensors.

**Figure 13:**
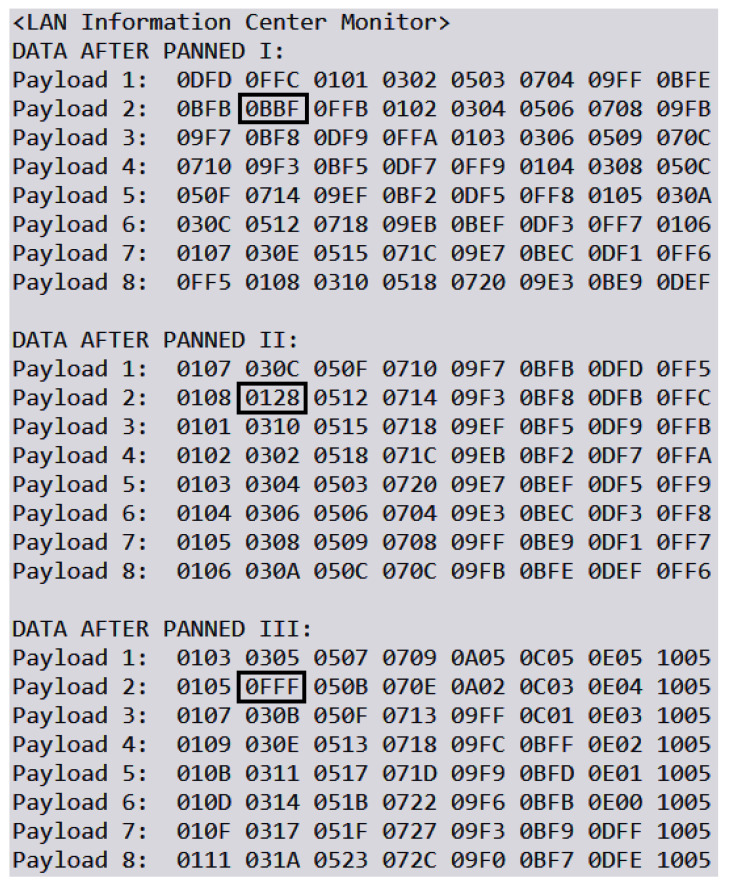
Heterogeneous data transmitted in LAN.

We simulate a man-in-the-middle (MITM) attack to evaluate the efficacy of our proposed security scheme in detecting unauthorized modifications to data. In this scenario, an attacker intercepts and randomly alters certain data blocks before they reach their intended destination. The security scheme should be able to identify these modifications and correct them. Figure 12 provides an example of a data packet transmitted from a Raspberry Pi to the server. The packet comprises eight blocks, each containing a 4-bit hexadecimal number that represents data collected from eight individual sensors. Upon initiating the simulated MITM attack, the altered packets in the secured overlay network are depicted in Figure 13. The tampered data blocks are highlighted with boxes to indicate unauthorized changes made by the attacker.

### 6.3. Security Analysis Metrics

We utilize the previously mentioned experimental setup to simulate data packet transmission in a SON. Specifically, we continuously transmit 10,000 data packets and assess the experimental results based on the following three key metrics:

Receive Accuracy: To evaluate the effectiveness of our proposed defense mechanism against data tampering attacks, we deliberately alter varying numbers of data blocks within each packet. The receive accuracy metric quantifies the proportion of data blocks received correctly. A higher receive accuracy implies a stronger defense against tampering.

Similarity: To evaluate the system’s ability to resist data stealing attacks, we compare randomly captured data packets during transmission with their original packets. We calculate similarity using the average longest common subsequence (LCS) and the Hamming distance. Lower similarity values indicate stronger defense capabilities against data-stealing attacks.

Numerical Offset: To evaluate the effectiveness of preventing attackers from concealing encryption patterns from potentially intercepted packets, we determine the numeric difference between data blocks in the original and encrypted packets. A larger numerical offset indicates a lower likelihood of attackers discerning encryption patterns through analysis of intercepted data.

### 6.4. Evaluation of Anti-Tampering Ability

**Figure 14:**
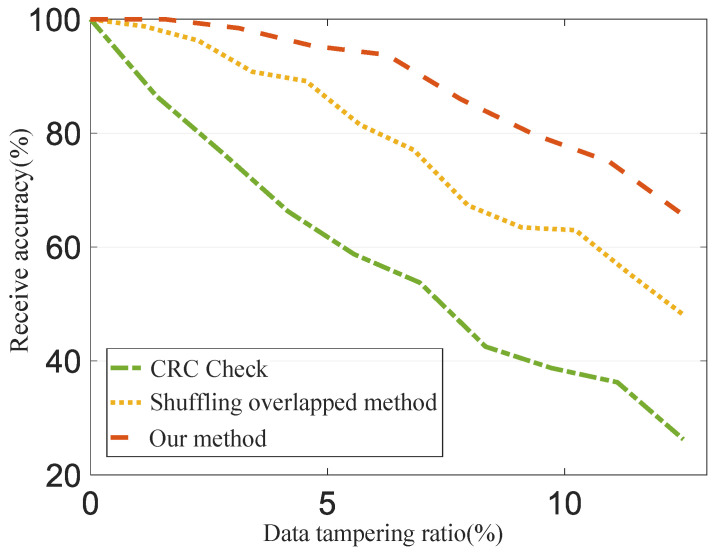
Evaluation of anti-tampering capability.

To evaluate the anti-tampering capability of our proposed DHR-based security system against data packet tampering attacks, we conduct experiments in which we randomly tamper with some data blocks in the data packet. We then analyze whether the sensor processing service of a SON received tampered data under different transmission methods. The results of these experiments are presented in Figure 14. Our method achieves 99.02% receive accuracy when 2% of data blocks are tampered with. The receive accuracy decreases when more than 10% of data blocks are tampered with. The experimental findings demonstrate that the anti-tampering ability of the CRC check method declines significantly as the data tampering rate increases. The shuffling overlapped method (SOM) enhances the anti-tampering ability to some extent, while our proposed method delivers the best performance. This is attributed to the combination of heterogeneity, redundancy, and dynamic adaptation within the DHR framework, rendering it highly resilient against a diverse array of attacks. Even if an attacker manages to compromise one or more data blocks, the remaining blocks can continue to provide protection, maintaining the overall security of the system.

### 6.5. Evaluation of Anti-Stealing Capability

**Figure 15:**
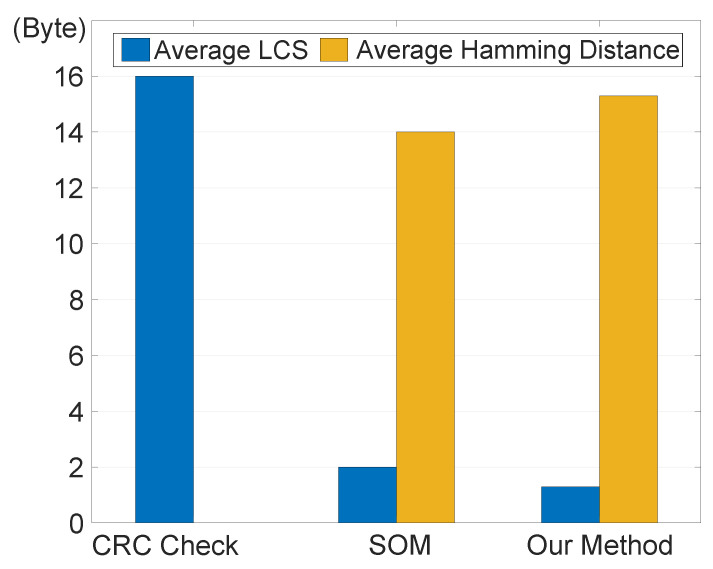
Data theft resistance under different transmission modes.

To analyze system’s efficacy against data stealing, we evaluate the average discrepancy between stolen data and its real value. We use Hamming distance and longest common subsequence (LCS) distance as measurement metrics. A greater Hamming distance signifies a larger dissimilarity between stolen and origin data, while a smaller LCS distance implies that attackers can obtain less information through stealing [36]. The experimental results displayed in Figure 15 reveal that with the CRC-16 check method, the LCS distance between the encrypted and original data is 16, while the minimum Hamming distance is 0. In contrast, our data encryption security scheme yields an average LCS distance of 1.3 and an average minimum Hamming distance of 15.3. These results surpass those of the SOM method, indicating that the ciphertext generated by our encryption module exhibits sufficient heterogeneity.

**Figure 16:**
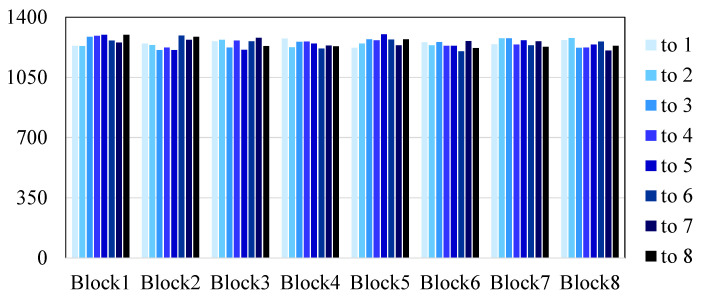
Frequency of heterogeneous data block offset.

Furthermore, we analyze the likelihood of attackers identifying encryption patterns from intercepted data. We achieve this by encrypting 10,000 packets from 3 sensors (i.e., METS sensor, CTD sensor, and DO sensor) and computing the block-shifting offset. The resulting distribution is depicted in Figure 16. It can be observed that the offset is uniformly distributed, without any recognizable pattern (i.e., Block1 has the same chance to be shifted to other blocks). This suggests that it is challenging for attackers to detect patterns and infer the original observation data. The findings demonstrate the efficacy of our security system in preventing data stealing. Consequently, it is considerably difficult for attackers to decipher the encryption rules of data packets through extended observation.

### 6.6. Evaluation on Side-Channel Attacks

**Figure 17:**
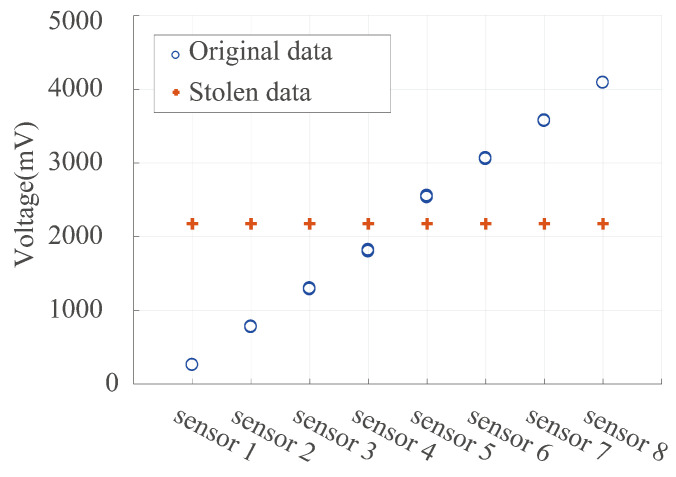
Comparison of theft data clustering centers and the observed data.

In light of potential side-channel attacks exploiting information gathered from a system’s physical characteristics, such as power consumption, our method stabilizes the amplitude of encrypted data. To validate this, we assessed the attacker’s ability to extract information. Specifically, we used the Pair-HMM method [37], to cluster 1000 pieces of encrypted data from each of the 8 sensors based on numerical values and compared it with the clustering of the original data. Figure 17 shows that when clustering the encrypted data produced by our method, it does not directly reveal the true value range of the original sensor data. The correlation coefficients between the stolen data and the original data resulted in value of [−0.2523, −0.4334, −0.5611, 0.1329, −0.3443, −0.9993, and −0.3444]. This confirms that our approach effectively increases the difficulty for attackers attempting physical attacks, such as side-channel attacks.

### 6.7. System Overhead Analysis

We select 8 sensors, each transmitting 16 bytes of observation data, to analyze data parity overhead during transmission. Typically, we employ our encryption method along with error-detecting codes (i.e., CRC-16, CRC-32, and SOM), hash mapping (i.e., MD5), and symmetric encryption (i.e., DES and AES) methods to encrypt the data on the Raspberry Pi, ensuring that all measurements are taken under the same configurations. Then, we conduct experiments to compare the resulting overhead length and execution time. Table 3 presents the execution time and overhead length results. We implement checksums and shifting operations using the NesC language on the TinyOS operating system. Processing times are measured using TinyOS’s LocalTimeMicroC components. In addition, our results demonstrate that our method outperforms CRC-16, CRC-32, and SOM. These methods require 52.8%, 92.2%, and 303.6% more execution time compared to our method, respectively. In terms of encryption algorithms, MD5, DES, and AES take 549.3×, 990.1×, and 20,520.5× more than our method, respectively. This highlights the efficiency of our method in lightweight and effective packet transmission without extra checksums while our DHR method includes redundant data transmission, which results in bandwidth consumption, it is crucial to notice that SONs are characterized by their wired connections and sufficient bandwidth resources. Consequently, our approach remains the preferred choice for enhancing information transmission security within SONs.

## 7. Conclusions

In this paper, we propose an innovative security information transmission approach for SONs based on dynamic heterogeneous redundancy theory. SONs consist of diverse devices that monitor the deep sea and communicate with onshore data centers. Due to extended data transmission distances and the network’s susceptibility to malicious attacks, ensuring data integrity is important. Our method employs dynamic keys to encrypt data blocks, generating multiple encrypted heterogeneous blocks for transmission. These dynamically changing encrypted blocks enhance confusion and diffusion of the original data by utilizing shuffle and shift operations. This significantly complicates attackers’ attempts to interpret or manipulate the data. Moreover, the redundancy within the multiple blocks assists in the identification and recovery of tampered data. Through empirical demonstrations in a minimal system, we validate the effectiveness of our approach in reducing data transmission errors and enhancing data integrity during transmission. In the future, we plan to apply our method to larger, more complex SONs to thoroughly evaluate their scalability and performance. Furthermore, we will certainly consider exploring the integration of our method with other security frameworks in our future work. For instance, we plan to leverage fuzzy neural networks to capture complex patterns and relationships within the data, aiming to enhance encryption quality and effectively manage high-dimensional data transmission.

## Figures and Tables

**Table 1:** Frequently used notations and descriptions.

Notations	Descriptions
$E_{i}\left(\right)$	The *i*-th encryption function
$E_{i}^{-1}\left(\right)$	The *i*-th decryption function
$A_{send}$	The set of all the plain text
$A_{recv}$	The set of all the ciphertext
*I*	Plain text to be sent
$I^{\prime }$	Ciphertext received

**Table 2:** The hardware’s detailed information.

Name	Model	Function	Configuration
METS Sensor	Franatech Classic METS [Reppenstedt, Germany]	Methane inspection	Measurement range of 50 nMol/L to 10 μMol/L
CTD Sensor	SAIV AS SD204 [Bergen, Norway]	Record seawater conductivity, salinity, temperature, depth, and sound speed (water density)	Salinity range: 0–40 ppt, Temperature range: −2–40 °C, Depth range: 500–6000 m
DO Sensor	Edaphic ES-O2-DW [Moorabbin, Australia]	Measure the oxygen in gas	Oxygen range: 0–20 mg/L
Raspberry Pi	Raspberry Pi 3 Model B [Shenzhen, China]	Simulate a junction box for encrypting collected sensor data	CPU: 64-bit quad-core, ARM Cortex-A53, Memory: 1 GB
Server	ThinkSystem SR558H [Beijing, China]	Simulate the operations center and onshore station to encrypt the information	CPU: Hygon C86 5280, Memory: 32 GB
Router	LS1008G V2 [Shenzhen, China]	Provide basic network topology	8 Ports, 10/100/1000 Mbps

**Table 3:** Overhead data comparison (bytes) and execution time (μs).

	Data	Overload	Overload (%)	Time
CRC-16 [6]	128	16	12.5	5.12 (+52.8%)
CRC-32 [38]	128	32	25	6.44 (+92.2%)
SOM [34]	128	48	37.5	13.52 (+303.6%)
MD5 [19]	128	128	100	1840.11 (×549.3)
DES [21]	128	64	50	3317.08 (×990.1)
AES [23]	128	128	100	68,743.82 (×20,520.5)
Our Method	128	256	200	3.35

## Data Availability

No new data were created or analyzed in this study. Data sharing is not applicable to this article.
